# Bariatric surgery prior to total knee arthroplasty is not associated with lower risk of revision: a register-based study of 441 patients

**DOI:** 10.1080/17453674.2020.1840829

**Published:** 2020-11-04

**Authors:** Perna Ighani Arani, Per Wretenberg, Johan Ottosson, Otto Robertsson, Annette W-Dahl

**Affiliations:** a Department of Orthopedics, Orebro University Hospital;; b Faculty of Medicine and Health, School of Medical Sciences, Örebro University, Örebro;; c Department of Surgery, Örebro University Hospital;; d Scandinavian Obesity Surgery Registry, Örebro;; e Lund University, Faculty of Medicine, Department of Clinical Sciences Lund, Orthopedics, Lund;; f The Swedish Knee Arthroplasty Register, Lund, Sweden

## Abstract

Background and purpose — Obesity is a considerable medical challenge in society. We investigated the risk of revision for any reasons and for infection in patients having total knee arthroplasty (TKA) for osteoarthritis (OA) within 2 years after bariatric surgery (BS) and compared them with TKAs without BS.

Patients and methods — We used the Scandinavian Obesity Surgery Registry (SOReg) and the Swedish Knee Arthroplasty Register (SKAR) to identify patients operated on in 2009–2019 with BS who had had primary TKA for OA within 2 years after the BS (BS group) and compared them with TKAs without prior BS (noBS group). We determined adjusted hazard ratio (HR) for the BS group and noBS group using Cox proportional hazard regression for revision due to any reasons and for infection. Adjustments were made for sex, age groups, and BMI categories preoperatively.

Results — 441 patients were included in the BS group. The risk of revision for infection was higher for the BS group with HR 2.2 (95% CI 1.1–4.7) adjusting for BMI before the TKA, while the risk of revision for any reasons was not statistically significant different for the BS group with HR 1.3 (CI 0.9–2.1). Corresponding figures when adjusting for BMI before the BS were HR 0.9 (CI 0.4–2) and HR 1.2 (CI 0.7–2).

Interpretation — Our findings did not indicate that BS prior to TKA was associated with lower risk of revision.

Overweight and obesity in society is a considerable medical challenge. It has been estimated that between 19% and 31% of the population in Europe is obese (BMI ≥ 30) and the proportion continues to increase (Krzysztoszek et al. 2019). Obesity is associated with a number of medical conditions such as type 2 diabetes, obstructive sleep apnea, cardiovascular diseases, dyslipidemia, fatty liver disease, and several cancers, contributing to a decline in both quality of life and life expectancy (Ng et al. 2014).

It has been shown that bariatric surgery (BS) is an effective method of achieving significant long-term weight loss for obese patients in comparison with nonsurgical interventions (Sjostrom 2013). BS results in significant improvement in multiple metabolic and cardiovascular diseases, reduction in new cancer development, and reduces risk for premature death in patients with BMI > 35 (Sjostrom et al. 2007, Adams et al. 2012, Sundbom et al. 2017). A significant improvement in health-related quality of life is also seen (Raoof et al. 2015, Kolotkin et al. 2018).

With the growing number of obese patients undergoing primary TKA, revision surgery among patients with obesity has increased over the past decades (Odum et al. 2016). Obesity has been described to negatively influence complications and mortality after TKA (Kerkhoffs et al. 2012, Electricwala et al. 2017, Tohidi et al. 2018). Several studies have shown an increased overall risk of revision after TKA in obese patients without looking at the specific reason for revision (Roche et al. 2018, Tohidi et al. 2018, Boyce et al. 2019). However, a recent study showed that obesity was associated with overall risk of revision and revision for infection, but not for revision for reasons other than infection (Sezgin et al. 2020).

There is no national guideline for BS in Sweden. Most hospitals follow the international accepted guidelines (Hubbard and Hall 1991). BS that may reduce BMI and subsequently comorbidities prior to a TKA could then be a beneficial measure to reduce the risk of revision. Nevertheless, this remains unclear considering previous studies (Inacio et al. 2014, Martin et al. 2015, Lee et al. 2018, McLawhorn et al. 2018) as well as in a recent systemic review (Gu et al. 2019).

Therefore, we evaluated the risk of revision for any reasons and for infection in OA patients having a TKA within 2 years after BS and compared them with TKAs without BS using data from the Scandinavian Obesity Surgery Registry (SOReg) and the Swedish Knee Arthroplasty Register (SKAR).

## Patients and methods

Patients who underwent BS in 2007–2019 were identified using the SOReg. From the SKAR we identified patients having primary TKA for OA in 2009–2019 within 2 years after the BS. From the SOReg we included all patients with primary gastric bypass and sleeve gastrectomy. The SOReg was established in 2007 and the SKAR in 1975. Both registers have high completeness and the correctness of data is validated with hospital records and found to be high (Hedenbro et al. 2015, SKAR 2019). The unique personal identification number that all Swedish citizens have was used when linking the datasets.

**Figure 1.**
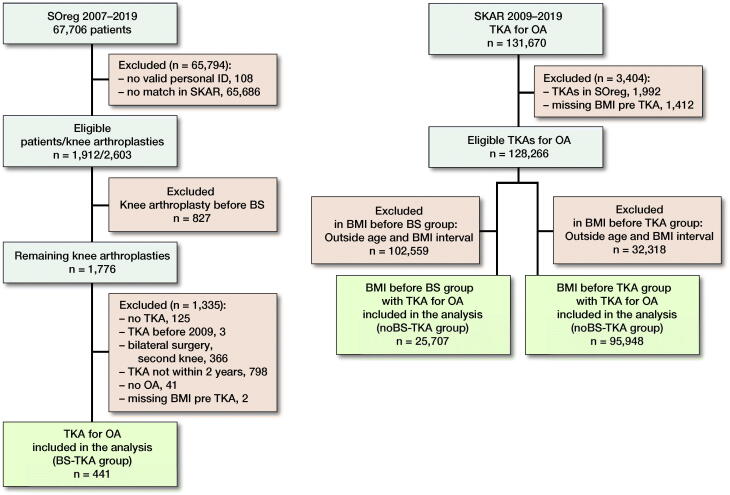
Flow-chart of the study.

In patients having BS before the TKA (BS group) we excluded the 2nd knee if both knees had a primary knee arthroplasty after the BS. Those with missing BMI before the TKA were excluded. In the cohort of TKAs without BS (noBS group) we excluded those with missing BMI before the TKA as well as the TKAs included in the SOReg (Figure 1).

We used 2 separate ways to compare the risk of revision in the BS group and the noBS group. In the 1st comparison we adjusted for the BMI prior to the BS in the BS group (BMI before BS), and in the 2nd comparison we adjusted for their BMI prior to the TKA (BMI before TKA) and compared them with the noBS TKA with comparable selections.

### TKA selections

In order to adjust for BMI before the BS in the BS group we selected the noBS group within the same BMI interval (BMI 32–63). For the analysis adjusting for BMI prior to the TKA in the BS group, we selected noBSs within the same BMI interval (range 16.9–50). In both analyses, we selected noBS TKAs within the same age interval as the BS TKA group had prior to the TKA surgery (range 39–76 years) (Figure 1).

BMI, age, and sex were obtained from the SKAR and SOReg. BMI was classified into 5 categories: underweight (BMI < 18.5), normal weight (BMI 18.5–24.9), overweight (BMI 25–29.9), obese (BMI 30–39.9), and morbidly obese (BMI ≥ 40). The ASA physical status classification was available from the SKAR. We decided not to adjust for the ASA classification in the primary analysis since BMI interferes with comorbidity (Owens et al. 1978) and BMI has also become a factor in deciding the ASA classification.

The outcome measures were revision for any reason and revision for suspected or verified infection. Revision is defined as a new operation in a previously resurfaced knee in which 1 or more of the components are exchanged, removed, or added (including arthrodesis and amputation) (SKAR 2019).

### Statistics

Adjusted hazard ratio (HR) was obtained for the BS groups and the noBS groups using Cox proportional hazards regressions for 2 endpoints: revision for any reasons over the whole period and revision for suspected or verified infection within 2 years after the primary TKA. Using BMI before the BS, adjustments were made for prior BS or no BS, sex, age groups (< 45, 45–54, 55–64, 65–74, and 75–84), and BMI category (30–39.9 and ≥ 40). Using BMI before the TKA adjustments were made for prior BS or no BS, sex, age groups (< 45, 45–54, 55–64, 65–74 and 75–84), and BMI category (< 18.5, 18.5–24.9, 25–29.9, 30–39.9, and ≥ 40). Sensitivity analyses were made including the ASA classification in the Cox regression analysis together with sex, age, and BMI when using BMI before the TKA with the 2 endpoints revision for any reasons and revision for suspected or verified infection. Results are reported as HRs with 95% confidence intervals (CIs). Statistical analyses were carried out using Stata version 15 (StataCorp, College Station, TX, USA).

### Ethics, funding, and potential conflicts of interest

The study was approved by the Swedish Ethical Review authority (2017/466). A research grant for the project was received from Region Örebro län. The authors have no conflicts of interest to report.

## Results

441 patients operated on with bariatric surgery prior to the TKA for OA (BS group) were included. When adjusting for BMI and age before the BS in the BS TKA group, the comparison was with 25,707 TKAs for OA not subject to BS but within the same age and BMI range as the patients in the BS group. When adjusting for the BMI and age prior to the TKA the BS group was compared with 95,948 TKAs for OA within the same age and BMI range.

The mean time between BS and TKA was 1.1 year (0.1–2). The mean BMI decreased from 43 (32–63) to 31 (17–50) between the BS and the TKA in the BS group. The mean follow-up for infection was 23 months in the BS group and 22 months in the noBS group and the follow-up for revision for any reason was 65 months in the BS group and 62 months in the noBS group.

### Adjusting for BMI prior to the BS

In the BS group the mean age was 55 years (37–74) and 76% were women. The mean BMI was 43 (32–63) before the BS (Table 1). The noBS group had a mean age of 65 years (39–76) and 64% were women. The mean BMI was 35 (32–50).

**Table 1.** Patient characteristics

	Restricting for BMI before BS		Restricting for BMI prior to TKA	
	BS group	noBS group	BS group	noBS group
Factor	n = 441	n = 25,707	n = 441	n = 95,948
Women ^a^	336 (76)	16,452 (64)	336 (76)	52,771 (55)
Age ^b^	55 (37–74)	65 (39–76)	57 (39–76)	66 (39–76)
BMI ^b^	43 (32–63)	35 (32–50)	35 (32–50)	29 (17–50)

**^a^** Count (%)

**^b^** Mean (range)

Using Cox proportional hazard regression, considering revision for any reasons as well as revisions for suspected or verified infection, we found similar risk, HR 1.2 (CI 0.7–2) and HR 0.9 (CI 0.4–2) respectively, for the BS TKA group as compared with the noBS TKA group without statistical significance (Table 2).

**Table 2.** Adjusted hazard ratios (aHR) for the risk of revision for infection and any reasons for revision when using BMI before the BS

	Revision for infection	Revision for any reasons
Variable	aHR (95% CI)	aHR (95% CI)
noBS group	Reference	Reference
BS group	0.9 (0.4–2.0)	1.2 (0.8–2.0)

### Adjusting for BMI prior to the TKA

The mean age of the BS group was 57 years (39–76) and 76% were women. The mean BMI was 31 (17–50) before the TKA (Table 1). In the noBS group the mean age was 65.8 years (39–76) and consisted of 55% women. The mean BMI was 29 (17–50).

Using Cox proportional hazard regression when analyzing revision for suspected or verified infection, we found a higher risk for the BS TKA group with HR 2.2 (CI 1.0–4.7) (Table 2). The risk of revision for any reasons was also higher for the BS TKA group with HR 1.3 (CI 0.9–2.1,), however without statistical significance (Table 3).

**Table 3.** Adjusted hazard ratios (aHR) for the risk of revision for infection and any reason for revision when using BMI before the TKA

	Revision for infection	Revision for any reasons
Variable	aHR (95% CI)	aHR (95% CI)
noBS group	Reference	Reference
BS group	2.2 (1.1–4.7)	1.3 (0.9–2.1)

The sensitivity analysis including ASA classification in the Cox regression analysis changed the results marginally: for the risk of revision for infection from HR 2.2 (CI 1.1–4.7) in the BS group to HR 2.0 (CI 1.0–4.3) with the noBS group as reference. Corresponding figures for the risk of revision for any reason was HR 1.3 (CI 0.9–2.1) and 1.3 (CI 0.8–2.0).

## Discussion

When analyzing the risk of revision for all reasons, we found no benefit for obese patients of having BS before the TKA when compared with TKAs without BS. This was independent of whether we adjusted for their BMI before the BS or before the TKA. However, the risk of revision due to suspected or verified infection was higher in patients having a BS before the TKA when using their BMI before the TKA, while the higher risk was not statistically significant when using the BMI before the BS in the BS group.

Our observational study based on national registers suffers from the limitations of this type of design. However, the data from SKAR and SOReg is prospectively collected, and both are national registers with a high completeness and quality (SOReg 2018, SKAR 2019). Another limitation is the relatively low number of 441 BS TKA cases. Nevertheless, this is the largest study of this type that has been performed.

When adjusting for BMI, for the BS group we used their BMI prior to the BS as well as prior to the TKA. The rationale was to make the comparison as if the BMI of the patients in the noBS group had been comparable to that of the BS group both before and after their weight loss. In order to make the groups more comparable we only included noBSs within the same BMI range as the BSs had on both occasions. Additionally, we only included the same age interval as in the BS TKA group, which on average was 13 years younger than the general TKA population in Sweden (SKAR 2019).

We set the cut-off time between BS and TKA as within 2 years to minimize the risk of other confounding health factors affecting outcome. In addition, time to TKA after BS has been suggested to be between 6 months and 2 years (Schwarzkopf et al. 2018).

The mean age in patients having BS in Sweden is just over 40 years and almost 80% are women (SOReg 2018). This may be reflected in the BS group were the patients were younger and the proportion of women was higher than in the general knee arthroplasty population in Sweden at the time of TKA. The Swedish bariatric surgery cohort has somewhat lower BMI (5–6 BMI units) and a lower percentage of comorbidity compared with most North American cohorts. Compared with some European cohorts the Swedish cohort have similar BMI and lower percentage of comorbidity (Poelemeijer et al. 2018).

We did not include information on comorbidity in the primary Cox regression analysis although it could affect the risk of revision. The SKAR includes the ASA classification while the SOReg includes the obesity surgery mortality risk score (OS-MRS). However, the patients undergoing elective surgery are assessed by both the surgeon and an anesthesiologist with optimization prior to surgery.

Earlier studies have demonstrated the relationship between BS prior to TKA regarding complications and outcome (Kulkarni et al. 2011, Inacio et al. 2014, Martin et al. 2015, Werner et al. 2015, Lee et al. 2018, McLawhorn et al. 2018).

The findings in previous studies investigating the risk of revision in patients having BS before the TKA as compared with patients not having BS are inconsistent. Martin et al. (2015) evaluated the BMI both before the BS and before the TKA in BS patients. They showed an increased risk of both reoperation and revision in 91 patients having a BS prior to the TKA (4 months to 11 years between the surgeries) as compared with a matched cohort of patients without BS. Infection was the most common reason for reoperation and revision. Also Lee et al. (2018) found an increased risk of revision in 70 patients having a BS before TKA within 2 years compared with a cohort of patients with chronic metabolic conditions, but found no increased risk of revision due to infection. Inacio et al. (2014) found no difference in risk of revision in 62 BS patients operated on with a TKA within 2 years after the BS as compared with 2,616 TKA patients without BS. McLawhorn et al. (2018) used propensity scores to match morbidly obese patients receiving and not receiving BS prior to TKA (2,636 TKA patients within each group) and found that BS did not reduce the risk of revision surgery. However, the time between the BS and the TKA was not presented. These studies included different selections of patients, BMI (i.e., BMI at the time for BS, BMI at the time for TKA, or both), selection of control group, time between the BS and TKA, and some have matched BS patients with TKA patients without BS for various variables. This makes comparison of these studies with ours difficult.

Despite the medical benefits in patients undergoing BS (Sjostrom et al. 2007, Adams et al. 2012, Sundbom et al. 2017), nutritional deficiencies may occur. Protein malnutrition is extremely uncommon with gastric bypass and sleeve gastrectomy but micronutrient deficiencies may be present (Xanthakos 2009, Bal et al. 2012). Also, bone demineralization and increased risk of fractures may occur after gastric bypass (Raoof et al. 2016, Axelsson et al. 2018). Our cohort includes gastric bypass and sleeve gastrectomy, the two most common types of BS today. Since the number of sleeve gastrectomies is low, no subgroup analysis was done.

To our knowledge, this is the 1st study to include a whole nation with close to complete registration of all TKA and BS procedures. In Sweden, there are no national guidelines for knee arthroplasty surgery. However, a national project with the intention to stop prosthetic-related infection after hip and knee arthroplasty surgery (the PRISS project) resulted in recommendations (2013), among others, for optimizing patients before surgery. The recommendations include, despite the absence of evidence, a suggestion that patients with BMI > 40 should be referred for help with preoperative weight loss (LÖF n.d.). While the proportion of obese individuals has increased in the Swedish population during the last decade (SCB n.d.) the proportion of patients with BMI ≥ 35 having a knee arthroplasty has decreased slightly (SKAR 2019). This may be a result of surgeons being more restrictive in operating on obese patients and/or recommending that obese patients consider BS or other measures to decrease their preoperative weight before the TKA surgery.

In conclusion, our findings indicate that having BS prior to TKA does not decrease the risk of revision. This information may be valuable when advising and scheduling obese OA patients before TKA surgery.
